# Changes in Epidemiology and Antibiotic Prescription of Influenza: Before and after the Emergence of COVID-19

**DOI:** 10.3390/ph17020181

**Published:** 2024-01-30

**Authors:** Mihai Aronel Rus, Bogdan Ghițoaica, Andrei Lucian Lazăr, Maria Ancuța Man, Violeta Tincuța Briciu, Monica Iuliana Muntean, Daniel Corneliu Leucuța, Mihaela Sorina Lupșe

**Affiliations:** 1 Department of Infectious Diseases, “Iuliu Hatieganu” University of Medicine and Pharmacy, 400348 Cluj-Napoca, Romania; ghitoaica.bogdan@elearn.umfcluj.ro (B.G.); monica.muntean@umfcluj.ro (M.I.M.);; 2 Teaching Hospital of Infectious Diseases, 400348 Cluj-Napoca, Romania; 3 Department of Medical Informatics and Biostatistics, Iuliu Hatieganu University of Medicine and Pharmacy, 400349 Cluj-Napoca, Romania

**Keywords:** influenza, COVID-19, post-pandemic, influenza management

## Abstract

Background: The appearance of COVID-19 had a major impact on healthcare and the epidemiology of other diseases. Following the cessation of non-pharmacologic interventions destined to limit the spread of COVID-19, influenza reemerged. The aim of this study was to compare the pre-pandemic influenza seasons with the influenza seasons after the emergence of the COVID-19 pandemic, and to identify differences in terms of clinical characteristics, risk factors, complications, outcomes, and antiviral and antibiotic treatments. Methods: We conducted a retrospective cohort study from the Teaching Hospital of Infectious Diseases database in Cluj-Napoca, Romania. We analyzed four pre-pandemic seasons and the seasons after the onset of COVID-19. We included adult patients hospitalized with confirmed influenza between October 2016 and August 2023. Variables such as age, sex, duration of hospitalization, severity, clinical manifestations, comorbidities, and Charlson comorbidity index were assessed. Results: A total of 941 patients were included in the analysis. The percentage of severe influenza was similar in both groups, but mortality from influenza was significantly lower after 2022. Virtually all patients were prescribed antivirals; antibiotic prescriptions decreased in the post-COVID-19 influenza seasons. Conclusion: The present study suggests that influenza seasons after 2022 had lower mortality and attenuated clinical presentation.

## 1. Introduction

The appearance of COVID-19 produced by the novel coronavirus SARS-CoV-2 had a major impact on healthcare, and the epidemiology of other communicable diseases. The public health measures intended to limit the spread of COVID-19 imposed in March 2020 also meant a sudden drop in the incidence of many other infections, including another potentially fatal respiratory illness—influenza. Influenza is caused by influenza type A or B virus; epidemics appear yearly during the cold season in temperate climate areas [1]. The reported incidence of influenza remained very low for more than one calendar year following the announcement of the COVID-19 lockdown, until January 2022; therefore, the 2020/2021 influenza season was peculiar, with only 168 confirmed cases in the EU region [2] and only 4 reported cases in Romania [3], and no reported deaths due to influenza in the EU region.

Influenza epidemiology is regularly monitored in Romania during the cold season. National data have shown that type A influenza was more prevalent than influenza type B in both pre-pandemic and post-pandemic seasons, with the exception of the 2017/2018 season [4,5,6,7].

In 2022, governments decided on the gradual cessation of non-pharmaceutical interventions, such as mandatory mask use, physical distancing, and staying at home, which represented for the general population a return to a “normal” post-pandemic status—and it also meant the re-emergence of influenza, with a four-fold rise in cases in the 2022/2023 season in Romania and Europe, compared to previous seasons [8,9].

The COVID-19 outbreak shed more light [10] on the need for the improved management of viral respiratory tract infections in terms of antiviral, antibiotic, and non-pharmacologic management, and protocols appeared quickly. In the same paradigm, the antiviral treatment of influenza has been established worldwide for many years, mainly with neuraminidase inhibitors, but the addition of antibiotics for the prevention or treatment of presumed bacterial superinfections in influenza is definitely a matter of debate, especially with respect to the concerning data on antibiotic resistance [11].

Patients vulnerable to developing severe influenza are considered to be patients > 65 years old with comorbidities such as diabetes mellitus, other chronic conditions such as asthma, chronic kidney disease, chronic heart failure, immunosuppression, and other conditions [12]. Considering that the new coronavirus pandemic increased awareness of vaccination and other measures to prevent the transmission of respiratory illnesses, we sought to evaluate whether epidemiologic changes to the known pattern have appeared, as data in this aspect are scarce. Studies comparing the epidemiology of influenza in relation to the COVID-19 pandemic are few and do not yet cover the 2022–2023 season [13]. We hypothesized that COVID-19, with all its challenges, improved our approach to respiratory tract infections and, implicitly, of influenza patients, as well as the outcomes.

The aim of the study was to compare the pre-pandemic influenza seasons with the influenza seasons that developed after the emergence of the COVID-19 pandemic and to identify differences in terms of clinical characteristics, risk factors, complications, and outcomes; a secondary aim was to assess trends in antiviral usage and the antibiotic treatment of influenza.

## 2. Results

### 2.1. Study Group Characterization

We identified a total of 941 patients to be included in the analysis, hospitalized for influenza in the Teaching Hospital of Infectious Diseases of Cluj-Napoca between 1 October 2016 and 30 August 2023, in accordance with the inclusion criteria. Patients were stratified into two major groups; before the appearance of COVID-19 (called Before-2020) and patients hospitalized after the appearance of COVID-19 (After-2022): 673 cases happened Before, while 268 cases were recorded after the appearance of the novel coronavirus. The percentage of severe influenza was similar in both groups—64.78% with 436 cases Before-2020 and 62.68% with 168 cases After-2022—but the frequency of intensive care unit (ICU) admittance lowered significantly After-2022, compared to the Before-2020 period: 23 cases (8.58%) and 109 cases (16.19%), respectively. Death was also significantly rarer After-2022, with a 3% fatality ratio, compared to 7% mortality of hospitalized cases Before-2020 (Table 1).

In both groups, a majority of patients were female—384 (57.05%) Before-2020 and 160 females (59.7%) in the After-2022 group, respectively. However, among the severe influenza cases and patients hospitalized in the ICU, numbers diverge: Before-2020, women accounted for 49% of severe cases and 41% of ICU cases, while After-2022, women accounted for 54% of severe cases and 52% of ICU cases. Group analysis is provided in Table 1.

Influenza A was the more frequent type in both groups and in all seasons studied, causing 531 hospitalizations (78.9%) in the group Before-2020 and 239 cases (89.17%) in the group After-2022. Approximatively one third of cases were diagnosed with PCR testing (269 cases), which also tested for respiratory syncytial virus (RSV); the rest of the cases were diagnosed with a rapid antigenic test for Influenza A and B virus, and the detection of coinfections with both influenza viruses, with RSV, or with COVID-19 were rare. A detailed distribution of cases related to the type of influenza is presented in Table 2.

The median age (range 18–95) was smaller in the After-2022 group, 59.5 years old, compared to the median age of 63 years old Before-2020; the two groups were identical in terms of median duration of hospitalization (6 days), average of Charlson comorbidity index (CCI) (3 points), and duration of symptoms until hospital presentation (3 days). The most frequent comorbidities were ischemic heart disease, diabetes mellitus, hypertension, chronic heart failure, and obesity. Asthma and obesity were more frequent After-2022 with statistical significance; other comorbidities present in the CCI and known to be related to severe influenza were highly similar between the two groups. There were six patients diagnosed with “flurona” (defined as concomitant infection with influenza and SARS-CoV-2 virus) and five patients with coinfection with RSV. The baseline characteristics and comorbidities of patients are presented in Table 3.

### 2.2. Analysis of Complicated/Severe Influenza

The occurrence of complicated or severe influenza, as defined by the WHO, was very similar between the two groups, with 436 (64.78%) cases Before-2020 and 168 (62.68%) cases After-2022, respectively. The patients with complicated influenza were significantly older than the non-severe ones, with a higher CCI, and, contrary to expectations, had a slightly prolonged delay to hospital presentation. Influenza type A was the most common cause of severe influenza. All evaluated comorbidities were more frequent in patients who developed complicated influenza, with statistical significance obtained for active cancer, ischemic heart disease, chronic kidney disease, COPD (chronic obstructive pulmonary disease), diabetes mellitus, and obesity.

Acute respiratory failure with the need for oxygen supplementation was the most frequent manifestation that granted the definition of complicated influenza, being similar between groups. The incidence of acute pneumonia with lobar infiltrates and acute renal failure was significantly lower in the After-2022 group. The need for CPAP or intubation was rarer in the After-2022 group, even though acute respiratory failure was more frequent. Stroke, atrial fibrillation, acute myocardial infarction, myocarditis, and myositis were not frequent complications, but nonetheless their incidence dropped in the After-2022 group. The number of pulmonary embolism cases grew significantly in influenza patients after the emergence of COVID-19. Table 4 and Table 5 present the manifestations found in patients with complicated/severe influenza in both study groups. Although interstitial pneumonia is not part of the definition of complicated influenza, Table 5 shows that its incidence increased in the After-2022 group.

We found a significant difference between the two study groups concerning ICU admittance. To investigate this association more thoroughly, we used multivariate logistic regression adjusted for confounders, revealing that the odds ratio of being hospitalized in the intensive care unit was 2.07 in the Before-2020 group; each increase in CCI was associated with an increase in OR of ICU admittance by 1.2. Influenza type A and male sex were also associated with an increased risk of ICU admittance; patients who radiologically developed pneumonia with infiltrates had an OR of ICU admittance of 8.76 (Table 6). The percentage of correct classification was 88.2%, and the area under the receiver–operator characteristic curve was 85.42 (81.95–88.88).

### 2.3. OR for Death Caused by Influenza

The evaluation of the fatal outcome showed that in the entire study group, there were 55 deaths: 47 (6.98%) happened before 2020, while 8 deaths (2.99%) occurred after the appearance of COVID-19, with a *p*-value of 0.018.

The patients at risk of fatal outcome had a median age of 75 years old, and were male patients with a CCI median of 6 points; most deaths were caused by influenza type A. Acute respiratory failure was the most common complication, frequently needing non-invasive or invasive ventilation, accompanied by pneumonia with lobar infiltrates and the need for ICU admittance; patients with fatal outcome were frequently diagnosed with sepsis or septic shock. Acute renal failure was not associated with the risk of dying in this study; also, myocarditis, myositis, encephalitis, and meningitis were rare diagnoses, and therefore without significant association with death; stroke and newly diagnosed atrial fibrillation were vascular complications correlated with death, but the number of cases was small. Conditions associated with risk of dying, with statistical significance, were active cancer, chronic kidney disease, congestive heart failure, dementia, ischemic heart disease, immunosuppression, leukemia, and also hypertension; interestingly, in this database, risk of death was not statistically associated with obesity, COPD, SOT recipients (solid organ transplantation), or diabetes mellitus. Table 7 represents manifestations and comorbidities of patients with fatal outcome, by comparison with patients discharged alive.

The death rate among hospitalized patients was not uniform across the seasons analyzed; it decreased after 2022, but it was also relatively small in the 2019–2020 influenza season. The rate of ICU admission also decreased in the influenza seasons after 2022, as shown in Table 8.

Multivariate logistic regression returned an odds ratio of dying from influenza before 2020 at roughly 2-fold higher than after the 2022 seasons; also, the odds of dying increased by 1.43 for each 1-point increase in the CCI score. Although influenza A was more frequent in patients with fatal outcome, the OR for Influenza A was only 0.48; among the five patients diagnosed with coinfection influenza + RSV, there were two deaths, both in the Before-2022 group—one being a 51-year-old female with acute leukemia, and one being an 83-year-old male with cardiovascular comorbidities, both with a CCI of 7. There were no fatalities among the flurona patients; the global percentage of correct classification of the test was 94.05%, with an AUC of 78.98 (73.36–84.6) (Table 9). Age > 65 was correlated with the risk of dying in univariate analysis, with an OR of 2.65, but considering that age is comprised in the CCI, it was not included separately in the regression model.

### 2.4. Use of Antivirals and Antibiotics in the Management of Influenza

With the exception of 1 patient in the study group, who declared oseltamivir allergy and did not receive the antiviral, all patients were treated with neuraminidase inhibitors; 1 patient received inhaled zanamivir, 1 patient received oseltamivir+baloxavir, and all the other 938 patients in the analysis received oseltamivir.

Antibiotic use for the management of patients hospitalized for influenza was reduced in the After-2022 group, with 76.87% of these subjects being prescribed an antibiotic; a minority of patients were prescribed Step 1 antibiotics of a narrower spectrum, and the most prescribed antibiotics were Step 2 antibiotics in both groups, mostly ceftriaxone, with an increase in the use of these antibiotics in the After-2022 group; the use of broad-spectrum antibiotics found in Step 3, mainly carbapenems, decreased after the appearance of the COVID-19 pandemic (Table 10). The association of two antibiotics comprised associations between Step 1 and Step 2 antibiotics (for example, the association of ceftriaxone with doxycycline, or ceftriaxone with macrolides), as is recommended in some cases of pneumonia.

As a rationale for antibiotic use, we could identify that this class of medication was prescribed to 80% of patients with a radiological aspect of interstitial pneumonia (*p* = 0.577); to 99.32% of patients with a radiological aspect of pneumonia with lobar infiltrates (*p* < 0.001); to 94.8% of patients with respiratory failure needing supplemental oxygen (*p* < 0.001); and to all patients hospitalized in the intensive care unit (*p* < 0.001) and all patients that were diagnosed with sepsis (*p* < 0.001). Step 2 antibiotics, mostly ceftriaxone, were prescribed most frequently, except for patients diagnosed with sepsis, which were initially prescribed Step 3 antibiotics, with broader coverage against Gram-positive and Gram-negative bacteria.

## 3. Discussion

This study involved a retrospective analysis of influenza hospitalization trends at the Teaching Hospital of Infectious Diseases in Cluj-Napoca, Romania. It compared data from the pre-COVID-19 period (2016–2020) with the period following February 2022, when regulations from the Romanian Ministry of Health allowed hospitalization in our unit of infections other than COVID-19.

We found that after the cessation of non-pharmaceutical interventions destined to prevent the transmission of COVID-19, influenza had an important rebound effect; in particular, the 2022–2023 season had the highest number of patients hospitalized for influenza (207 patients). This information is in accordance with European and national data showing the intense circulation of influenza in the 2022–2023 winter [8]. National sentinel surveillance data showed that in Romania in the 2022/2023 season, 3900 cases of influenza were reported, which was the highest number in the past 7 years analyzed. Also, European data revealed that in the 2022/2023 season there were 19,538 reported cases, the highest number in the previous 5 years [9]. Influenza type A was the most common finding in all seasons analyzed, both in severe and non-severe patients, and also in patients who had fatal outcomes. In this study, coinfection of flurona was not associated with bad outcomes, similar to another report [14], whereas coinfection with RSV returned bad outcomes with two deceased patients, but the number of patients with such diagnoses was small; RSV was able to produce severe infection with death in comorbid patients [15], but the low number of patients in this study does not render conclusions. In a study performed by Lee MK et al. [16], it was also found that coinfection with influenza and SARS-CoV-2, or with RSV, was rare.

Female sex was more prevalent among hospitalized cases in both study groups. In the After-2022 group, female sex remained more prevalent among severe influenza cases and those who needed ICU admittance, similar to other reports in the literature [17]. In the Before-2020 group, male sex was more prone to severe disease and ICU admittance. Similarly, in studies performed before COVID-19 by Martinez et al. [12], and Ezzine et al. [18], male sex was more prevalent among hospitalized patients and patients who died.

The two study groups were highly similar in terms of sex, duration of hospitalization, and comorbidities, including CCI. Differences we found were that patients hospitalized after 2022 were younger (63-year-old median age Before-2020 vs. 59.5-year-old median age After-2022), and asthma and obesity were more prevalent in this population, but asthma was still less frequent than in the 2009 flu pandemic caused by the AH1N2pdm09 strain [19]. The prevalence of obesity increased to 25% of hospitalized patients After-2022 in this database, which is more than other studies which addressed this [20,21]. The study groups were also very similar in the rates of severe cases (64.7% cases Before-2022, 62.69% After-2022). Interestingly, although the number of hospitalizations for influenza after 2022 was high, and the percentage of severe disease was highly similar between the study groups, the percentage of patients needing intensive care admission and the number of deaths were significantly lower in patients who developed influenza after 2022. One possible explanation could be the fact that even though the rate of respiratory failure needing oxygen supplementation increased after 2022, the rate of mechanical ventilation decreased, leading to the conclusion that the severity of the clinical syndrome was attenuated. In support of this conclusion is the finding that in cases occurring after 2022, the rate of pneumonia with lobar infiltrates decreased, while the rate of radiological interstitial aspect increased. Another possible explanation could rely on the finding that the median age of flu hospitalized after 2022 was lower. The prevalence of pneumonia with infiltrates of this study is lower than in other studies in the literature. Amira Jamoussi et al. found that pneumonia with infiltrates was present in 85% of their subjects [20], but their study was focused only on patients with severe acute respiratory infection. Kelsey M Sumner et al. [22] also identified a higher percentage of pneumonia in their group, but the definition of pneumonia was not provided.

Acute renal failure is another manifestation found relatively frequent in patients with severe disease in this study.

Comorbidities such as chronic kidney disease, COPD, dementia, hemiplegia, ischemic heart disease, and congestive heart failure had a highly similar prevalence between the two groups.

Severe/complicated influenza accounted for >60% of hospitalized patients in both study groups, as mentioned above.

Interestingly, pulmonary embolism was a complication encountered more often after 2022, even though the incidence was still low. The association between the flu and pulmonary embolism is not recognized to be frequent [23], but COVID-19 definitely paved the way for increased vigilance towards thrombo-embolic complications [24] and an active screening of this complication.

Pregnancy/post-natal status did not emerge as a significant risk factor for severe disease, probably due to high caution towards these patients, leading to hospitalization in many non-severe cases. The median age for the severe influenza patient was 69.5 years old and frequent comorbidities in these cases were obesity, diabetes mellitus, congestive heart failure, ischemic heart disease, chronic kidney disease, and also hypertension, findings in line with most of the literature [25].The risk of dying from influenza was about two times lower in the seasons after the emergence of the novel coronavirus, with a death rate of 2.99% among hospitalized adults; the result is similar to the finding of Xie Y et al. [26], who observed a death rate of 3.16% among hospitalized patients, across four months of the 2022–2023 influenza season in the USA. The median age of patients with fatal outcomes was 75 years old, with a median CCI of 6; considering the algorithm for calculating CCI, we can conclude that the patients at risk of dying had at least two comorbidities apart from advanced age. Chronic kidney disease, congestive heart failure, ischemic heart disease, diabetes mellitus, obesity, and cancer were frequent comorbidities in patients with fatal outcomes [27]. It is well stated in the literature that older patients with comorbidities are at the highest risk of death from influenza [28]. One notable exception beyond the CCI was an early post-natal patient who developed severe influenza and died, once again underlining the importance of vaccination in this population. Acute respiratory failure was by far the most frequent complication encountered in fatal cases, often in association with another very frequent complication—pneumonia; evolution with sepsis or septic shock were other frequent complications in patients who died. As influenza is a respiratory tract infection, respiratory failure and pneumonia are known in the literature as frequent features of severe cases [29]; the high percentage of patients diagnosed with sepsis or septic shock in this category underscores that older patients with comorbidities are a fragile category and influenza and hospitalization furthermore augment their frailty and unpredictable outcome.

We argue that a significant factor that led to decreased mortality in post-2022 seasons is related to the COVID-19 pandemic itself. A benefit that resulted from facing the challenges of the COVID-19 pandemic was that healthcare workers have better preparedness to address acute respiratory failure, which is the most common complication of severe influenza. Moreover, during the pandemic, hospital infrastructure was improved. Thus, improved patient management might have had an impact on mortality.

Influenza type A was most frequent in patients with fatal outcomes. However, in the multivariate analysis, type A flu did not emerge as a risk factor for dying due to the high incidence of this infection.

Almost all hospitalized patients received antiviral medication with neuraminidase inhibitors, with the exception of one patient who declared oseltamivir allergy. Related to antibiotic use, an optimistic finding of this analysis showed that antibiotic use, and most importantly, the use of broad-spectrum antibiotics, decreased in patients hospitalized after 2022, even though the use of third-generation cephalosporins slightly increased. There are, without a doubt, clinical situations when antibiotic use is necessary, such as presumed or proven bacterial pneumonia, sepsis, or other bacterial superinfections [30]. One possible explanation for lowering the use of broad-spectrum antibiotics could correlate with the lower mortality in this group, arguably suggesting attenuated forms in patients with severe infection. Another factor responsible for a decrease in antibiotic prescription is the development of government and hospital recommendations for limiting antibiotic usage. Romania has a high incidence of antimicrobial resistance [31], and therefore many limitations for antibiotic prescription have been imposed. One direct effect seen in this study is the significantly decreased usage of broad-spectrum antibiotics, mentioned here as Step 3. Although the tendency for antibiotic prescription has decreased in more recent seasons, it still exceeds the rate of patients with severe influenza by more than 10%, probably suggesting that better antibiotic stewardship is welcome.

Although this study provides detailed insight into the epidemiology of influenza, there are limitations to the present paper. First, it is a retrospective analysis starting from medical records available in the database of the Teaching Hospital of Infectious Diseases Cluj-Napoca, Romania. Therefore, information regarding influenza or COVID-19 vaccination was scarce and could not be considered for statistical analysis. Also, information regarding previous influenza or previous COVID-19 was not considered, which may have impacted the development of clinical syndromes and complications. The observational nature of the study precludes causation inference. Confounding can still happen, although we performed a multivariate logistic regression adjusting for many known factors, including the Charlson comorbidity index, which covers a broad spectrum of comorbidities. Second, the study includes data from a single center only, and therefore conclusions regarding the rate of hospitalizations in relation to influenza-like illness were not available. Third, the fact that the analysis considered different time periods, and we could say two different eras of medicine (before and after the emergence of COVID-19), probably impacted diagnostic and therapeutic approaches. Fourth, mortality data refer to patients who died in hospital, as the follow-up of patients after discharge was not feasible.

These study results can help us improve preparation for future flu seasons. We often see many hospitalizations in our unit during influenza seasons, which reflects how widely the flu virus is spreading in the country. There is a chance we might see large flu outbreaks in the future, and at the same time COVID-19 surges are possible. Therefore, it is very important for primary care physicians to be more alert about influenza. Also, we need to do better in getting people vaccinated against influenza in the upcoming years.

## 4. Materials and Methods

We conducted a retrospective cohort study using the database of the Teaching Hospital of Infectious Diseases from Cluj-Napoca, Romania, a tertiary care infectious diseases hospital. We analyzed four pre-pandemic influenza seasons—2016/2017, 2017/2018, 2018/2019, 2019/2020—and the seasons after the onset of COVID-19 pandemic—2022 and 2022/2023. The data were obtained from the medical records of the hospital database. The 2020/2021 season is not found in the database, and the 2021/2022 influenza season is not complete because, according to the regulations of the Romanian Ministry of Health, the Teaching Hospital of Infectious Diseases of Cluj-Napoca was classified as a COVID-19-dedicated hospital between March 2020 and February 2022; therefore, influenza patients were not admitted here during this time.

We included in the database adult patients who were hospitalized in the Teaching Hospital of Infectious Diseases Cluj-Napoca with confirmed influenza between 1 October 2016 and 30 August 2023. For inclusion in the database, we considered patients who presented to the Teaching Hospital of Infectious Diseases Cluj-Napoca with symptoms consistent for a respiratory tract infection. These patients were initially tested with a rapid antigenic test for influenza; when the rapid antigen test was negative, PCR testing was used. Only patients with positive tests for influenza were included in the analysis. The tests used were rapid antigen tests CerTest Biotec Influenza A+B^@^ (sensitivity and specificity > 99%, according to manufacturer data) and VitroBioChem Influenza A+B^@^ (sensitivity for detecting type A influenza 87.2%; sensitivity for type B influenza 92.5%; specificity for type A influenza 94.5%; and specificity for type B influenza 97.5%, according to manufacturer data) or molecular test GeneXpert-Cepheid^@^ (98.9% sensitivity for influenza A; 98.4% sensitivity for influenza B; 97.5% specificity for influenza A; 99.3% specificity for influenza B) and DiagCORE-Qiagen^@^ (97.84% sensitivity and 99.45% specificity). After the appearance of SARS-CoV-2 infections, all patients were also tested for COVID-19.

For inclusion in the database, subjects were initially screened with ICD10 coding, using codes J10.0, J10.1, J10.8, J11.0, J11.1, and J11.8. Patients < 18 years-old, patients with influenza-like illness (ILI) without influenza testing, or those with negative tests were excluded from the analysis; also excluded from the analysis were patients whose reason for admittance and management was a different infection unrelated to influenza, such as *Clostridioides difficile* colitis, endocarditis, or urinary tract infection with no or mild respiratory symptoms. The case description was according to WHO recommendations.

We retrieved information regarding antibiotic use from the database, which was stratified as Step 1 antibiotics for respiratory tract infections (macrolides, second generation cephalosporins, doxycycline, aminopenicillins) (Step 1 antibiotics are the ones with a narrower spectrum of activity and usually prescribed orally); Step 2 antibiotics, which are generally used in the management of pneumonia (third generation cephalosporins, respiratory fluoroquinolones) and were prescribed intravenously; and Step 3, which are broad-spectrum antibiotics (carbapenems, piperacillin/tazobactam, vancomycin, linezolid, ciprofloxacin, levofloxacin, colistin), reserved for in-hospital intravenous use, after the failure of other classes of antibiotics. For a better understanding, we assumed Step 1, 2 and 3 as the first, second, and third line of antibiotics.

A complicated influenza case was described, according to the WHO [32] as a case who developed infectious complications such as pneumonia, respiratory failure, including ARDS, sepsis, septic shock, encephalitis, or other influenza-related complications such as stroke, myocardial infarction, fibrillation, myocarditis, myositis, and acute renal failure.

The study has the approval of the Ethics Committee of the Teaching Hospital of Infectious Diseases Cluj-Napoca.

For each subject, we extracted variables such as age, sex, duration of hospitalization, delay to admission, intensive care unit (ICU) admission, clinical manifestations, comorbidities (hypertension, diabetes, obesity = BMI ≥ 30 kg/m^2^, immunosuppression, chronic respiratory failure, chronic kidney disease, anemia, heart failure, rheumatological conditions), radiological aspect, complications (acute respiratory failure, pneumonia, acute renal failure, myocardial infarction, atrial fibrillation, pulmonary embolism, stroke, meningitis/encephalitis, sepsis), treatment, and status at discharge. The immunosuppressed patients were defined as patients with HIV infection, hematological malignancy, solid tumor with ongoing treatment, neutropenia, and transplant recipients. CCI was assessed. We also recorded data regarding influenza type A and B.

Data collection was performed with Microsoft Excel, and statistical analysis with R environment for statistical computing. Categorical data were presented as counts and percentages. Non-normally distributed quantitative data were presented as medians and the first and the third quartiles. Comparisons between two groups concerning categorical variables were performed with the Chi-squared test or Fisher exact test. Comparisons between two groups concerning non-normally distributed quantitative variables were performed with the Wilcoxon rank-sum test. To explore the relationship between Before-2020 and After-2022 and ICU admittance or death, we fitted multiple logistic regression models, including the study groups, and adjusted for known manually chosen confounders (CCI, sepsis, pneumonia with lobal infiltrates, sex, type A influenza, and coinfection). For all models, the Hosmer and Lemeshow goodness-of-fit test was used. The assumption of multicollinearity was verified with the variance inflation factor and correlations. The models were reported using odds ratios, with 95% confidence intervals, *p*-values, and their classification ability with area under the receiver operator characteristic (AUROC) curve.

## Figures and Tables

**Table 1:** Group analysis of study population and sex distribution.

Group	No. of Patients	Severe Influenza	ICU Admittance	Deaths
Before-2020	673 (384 F—57.05%)	436 (214 F—49.08%)	109 (45 F—41.28%)	47 (20 F—42.55%)
After-2022	268 (160 F—59.7%)	168 (91 F—54.16%)	23 (12 F—52.17%)	8 (4 F—50%)

F, female; ICU, intensive care unit.

**Table 2:** The seasonal distribution of patients hospitalized for influenza between 2016 and 2023.

Group	Season	No. of Pts.	Influenza A	Influenza B	Coinfection
**Before-2020**	2016/2017	81	77 (95.06%)	3 (3.7%)	1 pt A+RSV (1.23%)
	2017/2018	205	116 (56.6%)	84 (40.9%)	5 pts A+B (2.4%)
	2018/2019	202	187 (92.5%)	9 (4.45%)	2 pts A+B (0.99%) 4 pts A+RSV (1.98%)
	2019/2020	185	151 (81.62%)	34 (18.37%)	0
	**All 2016–2020**	673	531 (78.9%)	130 (19.31%)	12 (1.78%)
**After-2022**	2022	61	57 (93.44%)	2 (3.27%)	1 pt A+COVID-19 (1.63%) 1 pt B+COVID-19 (1.63%)
	2022/2023	207	182 (87.92%)	19 (9.17%)	2 pts A+B (0.96%) 3 pts A+COVID19 (1.44%) 1 pt B+COVID-19 (0.48%)
	**All 2022–2023**	268	239 (89.17%)	21 (7.83%)	8 (2.98%)
**Total**		941	770 (81.82%)	151 (16.04%)	20 (2.12%)

Pt, patient; RSV, respiratory syncytial virus; A, type A influenza; B, type B influenza.

**Table 3:** Characteristics of hospitalized influenza patients.

Variables	Before-2020 (*n* = 673)	After-2022 (*n* = 268)	*p*-Value
Age (years), median (IQR)	63 (42–76)	59.5 (36.75–75.25)	0.194
Sex (F), *n* (%)	384 (57.06)	160 (59.7)	0.459
CCI, median (IQR)	3 (1–5)	3 (0–5)	0.041
Duration of hospitalization (days), median (IQR)	6 (5–9)	6 (4–8)	0.029
Days before hospital presentation, median (IQR)	3 (2–5)	3 (2–5)	0.023
Severe influenza, *n* (%)	436 (64.78)	168 (62.69)	0.545
ICU admission, *n* (%)	109 (16.2)	23 (8.58)	0.002
Death, *n* (%)	47 (6.98)	8 (2.99)	0.018
***Comorbidities***			
Active cancer, *n* (%)	40 (5.94)	10 (3.73)	0.172
Asthma, *n* (%)	39 (5.79)	26 (9.7)	0.033
Connective tissue disease, *n* (%)	17 (2.53)	3 (1.12)	0.177
Chronic kidney disease, *n* (%)	58 (8.62)	24 (8.96)	0.869
Chronic hepatitis, *n* (%)	24 (3.57)	11 (4.1)	0.694
Congestive heart failure, *n* (%)	117 (17.38)	41 (15.3)	0.44
COPD, *n* (%)	88 (13.08)	43 (16.04)	0.235
Dementia, *n* (%)	33 (4.9)	16 (5.97)	0.506
Diabetes mellitus, *n* (%)	160 (23.77)	59 (22.01)	0.564
Hemiplegia, *n* (%)	32 (4.75)	12 (4.48)	0.856
Hypertension, *n* (%)	370 (54.98)	144 (53.73)	0.729
History of myocardial infarction, *n* (%)	36 (5.35)	19 (7.09)	0.304
Ischemic heart disease, *n* (%)	178 (26.45)	69 (25.75)	0.825
Immunosuppressed, *n* (%)	82 (12.18)	22 (8.21)	0.079
Leukemia, *n* (%)	9 (1.34)	2 (0.75)	0.738
Lymphoma, *n* (%)	3 (0.45)	1 (0.37)	1
Liver cirrhosis, *n* (%)	15 (2.23)	3 (1.12)	0.262
Obesity, *n* (%)	129 (19.17)	68 (25.37)	0.035
Peripheral vascular disease, *n* (%)	59 (8.77)	18 (6.72)	0.3
Pregnant/post-natal, *n* (%)	30 (7.87)	12 (7.45)	0.867
SOT recipient, *n* (%)	6 (0.89)	1 (0.37)	0.68

SOT—solid organ transplantation; COPD—chronic obstructive pulmonary disease; IQR—interquartile range.

**Table 4:** Characterization of the patients with severe influenza.

	Severe Influenza (*n* = 604)	Non-Severe Influenza (*n* = 337)	*p*-Value
Age, median (IQR)	69.5 (58–80)	36 (29–59)	<0.001
Charlson comorbidities index, median (IQR)	4 (3–6)	0 (0–2)	<0.001
Duration of hospitalization, median (IQR)	7 (6–10)	4 (3–6)	<0.001
Days before hospital presentation, median (IQR)	3 (2–5)	2 (1–4)	<0.001
Influenza type, *n* (%)	A: 524 (86.75) A+B: 6 (0.99) B: 74 (12.25)	A: 255 (75.67) A+B: 3 (0.89) B: 79 (23.44)	<0.001
***Comorbidities***			
Active cancer, *n* (%)	40 (6.62)	10 (2.97)	0.017
Asthma, *n* (%)	46 (7.62)	19 (5.64)	0.251
Connective tissue disease, *n* (%)	16 (2.65)	4 (1.19)	0.136
Chronic kidney disease, *n* (%)	81 (13.41)	1 (0.3)	<0.001
Chronic hepatitis, *n* (%)	23 (3.81)	12 (3.56)	0.848
Congestive heart failure, *n* (%)	146 (24.17)	12 (3.56)	<0.001
COPD, *n* (%)	119 (19.7)	12 (3.56)	<0.001
Dementia, *n* (%)	46 (7.62)	3 (0.89)	<0.001
Diabetes mellitus, *n* (%)	181 (29.97)	38 (11.28)	<0.001
Hemiplegia, *n* (%)	40 (6.62)	4 (1.19)	<0.001
Hypertension, *n* (%)	426 (70.53)	88 (26.11)	<0.001
History of myocardial infarction, *n* (%)	47 (7.78)	8 (2.37)	<0.001
Ischemic heart disease, *n* (%)	220 (36.42)	27 (8.01)	<0.001
Immunosuppressed, *n* (%)	72 (11.92)	32 (9.5)	0.255
Leukemia, *n* (%)	8 (1.32)	3 (0.89)	0.755
Lymphoma, *n* (%)	1 (0.17)	3 (0.89)	0.134
Liver cirrhosis, *n* (%)	13 (2.15)	5 (1.48)	0.473
Obesity, *n* (%)	161 (26.66)	36 (10.68)	<0.001
Peripheral vascular disease, *n* (%)	71 (11.75)	6 (1.78)	<0.001
Pregnant/post-natal, *n* (%)	9 (2.9)	33 (14.22)	<0.001
SOT recipient	4 (0.66)	1 (0.3)	0.66

SOT—solid organ transplantation; COPD—chronic obstructive pulmonary disease; IQR—interquartile range.

**Table 5:** Group manifestations of complicated/severe influenza.

Variables	Before-2020 (*n* = 673)	After-2022 (*n* = 268)	*p*-Value
Acute respiratory failure, *n* (%)	349 (51.86)	151 (56.34)	0.213
- CPAP, *n* (%)	139 (20.65)	41 (15.3)	0.059
- Intubation, *n* (%)	58 (8.62)	9 (3.36)	0.005
Acute pneumonia with infiltrates, *n* (%)	225 (33.43)	70 (26.12)	0.029
* Interstitial pneumonia *, *n* (%)	164 (24.37)	107 (39.93)	<0.001
Acute myocardial infarction, *n* (%)	4 (0.59)	1 (0.37)	1
Acute renal failure, *n* (%)	172 (25.50)	48 (17.91)	0.012
Acute myocarditis, *n* (%)	2 (0.3)	0 (0)	1
Acute myositis, *n* (%)	3 (0.45)	1 (0.37)	1
Encephalitis/meningitis, *n* (%)	5 (0.74)	0 (0)	0.329
Newly diagnosed atrial fibrillation, *n* (%)	15 (2.23)	5 (1.87)	0.727
Pulmonary embolism, *n* (%)	3 (0.45)	9 (3.36)	0.001
Sepsis, *n* (%)	50 (7.43)	17 (6.34)	0.559
Septic shock), *n* (%)	21 (3.12)	6 (2.24)	0.465
Stroke during hospitalization, *n* (%)	4 (0.59)	1 (0.37)	1

* Interstitial pneumonia itself was not considered as a reason for classification as severe influenza, but is shown in table for comparison with consolidation pneumonia; CPAP—continuous positive airwave pressure.

**Table 6:** Multivariate logistic regression predicting ICU admittance for all study groups, adjusted for Charlson comorbidity index, type A influenza, sex, sepsis, and pneumonia with lobar infiltrates.

Variables	OR Adjusted	(95% CI)	*p*
Before 2020 vs. after 2022	2.07	(1.22–3.65)	0.009
Charlson comorbidity index	1.2	(1.1–1.31)	<0.001
Type A influenza	1.71	(0.89–3.49)	0.118
Coinfection with other respiratory viruses	1.13	(0.23–4.23)	0.868
Male sex	1.18	(0.76–1.83)	0.45
Sepsis	5.23	(2.86–9.69)	<0.001
Pneumonia with lobar infiltrates	8.76	(5.52–14.3)	<0.001

ICU, intensive care unit; OR, odds ratio; CI, confidence interval.

**Table 7:** Status at discharge of patients hospitalized for influenza.

	Deceased (*n* = 55)	Alive (*n* = 886)	*p*-Value
Age, median (IQR)	75 (62.5–84)	61 (38–75)	<0.001
Charlson comorbidities index, median (IQR)	6 (5–7)	3 (0–5)	<0.001
Sex (F), *n* (%)	24 (43.64)	520 (58.69)	0.028
Influenza type, *n* (%)	A: 39 (70.9) A+B: 0 (0) A+RSV: 2 (3.63) B: 14 (25.45) Flurona: 0 (0)	A: 731 (82.5) A+B: 9 (1.01) A+RSV: 3 (0.33) B: 137 (15.46) Flurona: 6 (0.67)	0.13
***Complications***			
Acute respiratory failure, *n* (%)	53 (96.36)	447 (50.45)	<0.001
CPAP, *n* (%)	34 (61.82)	146 (16.48)	<0.001
Intubation, *n* (%)	35 (63.64)	32 (3.61)	<0.001
Acute pneumonia with lobar infiltrates, *n* (%)	46 (83.64)	249 (28.1)	<0.001
Interstitial pneumonia, *n* (%)	4 (7.27)	267 (30.14)	<0.001
Acute myocardial infarction, *n* (%)	1 (1.82)	4 (0.45)	0.261
Acute renal failure, *n* (%)	14 (25.45)	707 (79.8)	<0.001
Acute myocarditis, *n* (%)	0 (0)	2 (0.23)	1
Acute myositis, *n* (%)	0 (0)	4 (0.45)	1
Encephalitis/meningitis, *n* (%)	1 (1.82)	4 (0.45)	0.261
ICU admittance, *n* (%)	46 (83.64)	86 (9.71)	<0.001
Newly diagnosed atrial fibrillation, *n* (%)	6 (10.91)	14 (1.58)	<0.001
Pulmonary embolism, *n* (%)	1 (1.82)	11 (1.24)	0.517
Sepsis, *n* (%)	17 (30.91)	50 (5.64)	<0.001
Stroke during hospitalization, *n* (%)	3 (5.45)	2 (0.23)	0.002
Septic shock, *n* (%)	23 (41.82)	4 (0.45)	<0.001
***Comorbidities***			
Active cancer, *n* (%)	9 (16.36)	41 (4.63)	0.002
Asthma, *n* (%)	3 (5.45)	62 (7)	1
Connective tissue disease, *n* (%)	3 (5.45)	17 (1.92)	0.106
Chronic kidney disease, *n* (%)	13 (23.64)	69 (7.79)	<0.001
Chronic hepatitis, *n* (%)	2 (3.64)	33 (3.72)	1
Congestive heart failure, *n* (%)	17 (30.91)	141 (15.91)	0.004
COPD, *n* (%)	11 (20)	120 (13.54)	0.18
Dementia, *n* (%)	8 (14.55)	41 (4.63)	0.006
Diabetes mellitus, *n* (%)	18 (32.73)	201 (22.69)	0.087
Hemiplegia, *n* (%)	5 (9.09)	39 (4.4)	0.174
Hypertension, *n* (%)	38 (69.09)	476 (53.72)	0.026
History of myocardial infarction, *n* (%)	7 (12.73)	48 (5.42)	0.036
Ischemic heart disease, *n* (%)	26 (47.27)	221 (24.94)	<0.001
Immunosuppressed, *n* (%)	13 (23.64)	91 (10.27)	0.002
Leukemia, *n* (%)	3 (5.45)	8 (0.9)	0.022
Lymphoma, *n* (%)	0 (0)	4 (0.45)	1
Liver cirrhosis, *n* (%)	3 (5.45)	15 (1.69)	0.083
Obesity, *n* (%)	12 (21.82)	185 (20.88)	0.868
Peripheral vascular disease, *n* (%)	7 (12.73)	70 (7.9)	0.204
Pregnant/post-natal, *n* (%)	1 (3.85)	41 (7.95)	0.711
SOT recipients, *n* (%)	0 (0)	5 (0.56)	1

CPAP, continuous positive airwave pressure; COPD, chronic obstructive pulmonary disease; SOT, solid organ transplantation.

**Table 8:** Rates of death and ICU admittance among patients hospitalized for influenza across studied seasons.

Season:	2016–2017 (*n* = 81)	2017–2018 (*n* = 205)	2018–2019 (*n* = 202)	2019–2020 (*n* = 185)	2022 (*n* = 61)	2022–2023 (*n* = 207)	*p*-Value
Death, *n* (%)	10 (12.35)	19 (9.27)	15 (7.43)	3 (1.62)	1 (1.64)	7 (3.38)	<0.001
ICU admittance, *n* (%)	21 (25.93)	30 (14.63)	32 (15.84)	26 (14.05)	1 (1.64)	22 (10.63)	0.001

ICU, intensive care unit.

**Table 9:** Multivariate logistic regression predicting death in relation to group, adjusted for Charlson comorbidities index, type of flu, and coinfection with RSV.

Variables	OR Adjusted	(95% CI)	*p*
Before-2020 vs. After-2022	2.01	(0.95–4.75)	0.085
Charlson comorbidity index	1.43	(1.29–1.59)	<0.001
Type A Influenza	0.48	(0.25–0.97)	0.034
Coinfection with other respiratory viruses	1.94	(0.3–7.42)	0.395

OR, odds ratio; CI, confidence interval.

**Table 10:** Antibiotic use in the management of influenza patients.

Variables	Before-2020 (*n* = 673)	After-2022 (*n* = 268)	*p*-Value
Antibiotics prescribed, *n* (%)	558 (82.91)	206 (76.87)	0.032
Association of 2 antibiotics, *n* (%)	195 (28.97)	60 (22.39)	0.04
Step 1 antibiotics, *n* (%)	165 (24.52)	54 (20.15)	0.152
Step 2 antibiotics, *n* (%)	355 (52.75)	158 (58.96)	0.084
Step 3 antibiotics, *n* (%)	133 (19.76)	24 (8.96)	<0.001

Step 1 antibiotics—macrolides, second generation cephalosporins, doxycycline, aminopenicillins; Step 2 antibiotics—third generation cephalosporins, moxifloxacin; Step 3 broad spectrum antibiotics—carbapenems, piperacillin/tazobactam, vancomycin, linezolid, ciprofloxacin, levofloxacin, colistin.

## Data Availability

Data used was retrieved from the database of the Teaching Hospital of Infectious Disease, Cluj-Napoca, Romania. The dataset is not public, but may be made available on request; please contact the corresponding authors.
